# Correction: Alamri et al. Enhancement of the Protective Activity of Vanillic Acid against Tetrachloro-Carbon (CCl_4_) Hepatotoxicity in Male Rats by the Synthesis of Silver Nanoparticles (AgNPs). *Molecules* 2022, *27*, 8308

**DOI:** 10.3390/molecules30010143

**Published:** 2025-01-02

**Authors:** Eman S. Alamri, Haddad A. El Rabey, Othman R. Alzahrani, Fahad M. Almutairi, Eman S. Attia, Hala M. Bayomy, Renad A. Albalwi, Samar M. Rezk

**Affiliations:** 1 Department of Nutrition and Food Science, University of Tabuk, Tabuk 47512, Saudi Arabia; 2 Biochemistry Department, Faculty of Science, University of Tabuk, Tabuk 47512, Saudi Arabia; 3 Bioinformatics Department, Genetic Engineering and Biotechnology Research Institute, University of Sadat City, Sadat City 32897, Egypt; 4 Department of Biology, University of Tabuk, Tabuk 47512, Saudi Arabia; 5 National Nutrition Institute, Ministry of Health, Cairo 4262114, Egypt; 6 Department of Food Science and Technology, Damanhour University, Damanhour 22511, Egypt; 7 Clinical Nutrition Department, Mahalla Hepatology Teaching Hospital, El-Mahalla El-Kubra 4260010, Egypt

In the original publication [1], there was a mistake in Figure 7E as published. Figure 7A was copied by mistake and inserted as Figure 7E. The corrected Figure 7 appears below. The authors state that the scientific conclusions are unaffected. This correction was approved by the Academic Editor. The original publication has also been updated.

**Figure 7:**
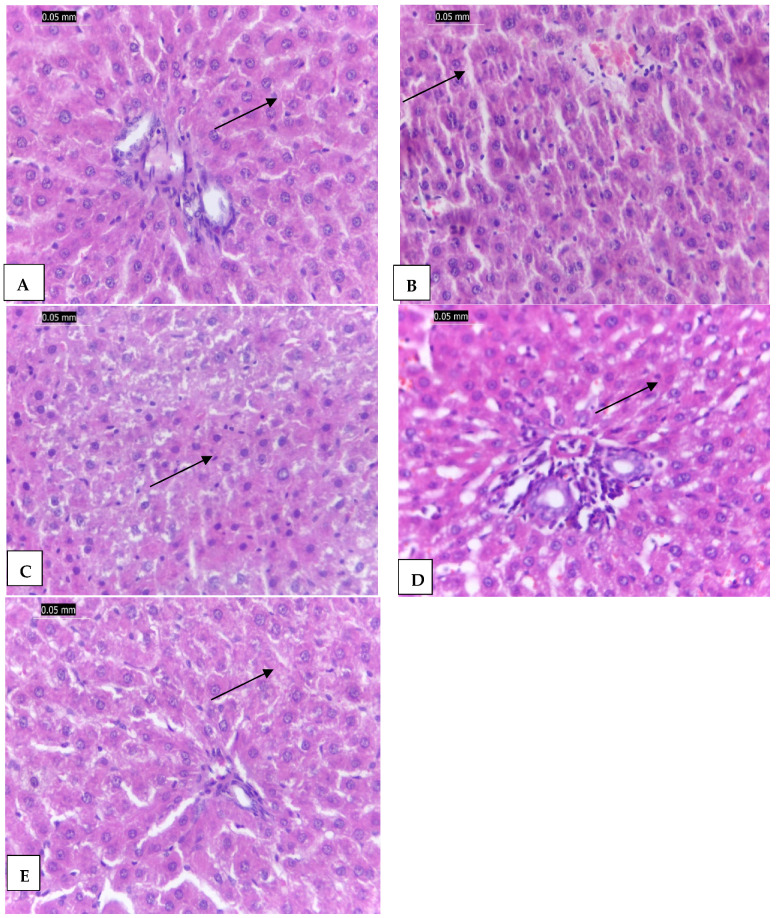
(**A**): The control negative group (G1) showing normal hepatic tissues architecture without any necrosis or inflammation (arrow), (**B**): The CCl_4_ treated group (G2) showing distorted hepatic plates, fatty hepatocytes (arrow), and dense portal tract mononuclear infiltrate, (**C**): G3 treated with vanillic acid showing mild inflammation (arrow), (**D**): The G4 group treated with vanillic AgNPs showing mild inflammation to nearly normal hepatic tissues (arrow), and (**E**): The silymarin treated group (G5) showing nearly normal hepatic tissues (arrow). (H & E × 400).
