# Supplementary material for: Enhancement of the Protective Activity of Vanillic Acid against Tetrachloro-Carbon (CCl4) Hepatotoxicity in Male Rats by the Synthesis of Silver Nanoparticles (AgNPs)
Source: Molecules. 2022 Nov 28;27(23):8308. doi: 10.3390/molecules27238308 (PMC9737075; doi:10.3390/molecules27238308)
Supplement: Supplementary file 1 [file molecules-27-08308-s001.zip › molecules-1995782-supplementary.pdf]

Supplementary material

Enhancement of the Protective Activity of Vanillic Acid against Tetrachloro–Carbon (CCl_4) Hepatotoxicity in Male Rats by the Synthesis of Silver Nanoparticles (AgNPs)

Eman S. Alamri [1], Haddad A. El Rabey [2,3,*], Othman R. Alzahrani [4], Fahad M. Almutairi [2], Eman S. Attia [5], Hala M. Bayomy [1,6], Renad A. Albalwi [1] and Samar M. Rezk [7]

[1] Department of Nutrition and Food Science, University of Tabuk, Tabuk 47512, Saudi Arabia
[2] Biochemistry Department, Faculty of Science, University of Tabuk, Tabuk 47512, Saudi Arabia
[3] Bioinformatics Department, Genetic Engineering and Biotechnology Research Institute, University of Sadat City, Sadat City 32897, Egypt
[4] Department of Biology, University of Tabuk, Tabuk 47512, Saudi Arabia
[5] National Nutrition Institute, Ministry of Health, Cairo 4262114, Egypt
[6] Department of Food Science and Technology, Damanhour University, Damanhour 22511, Egypt
[7] Clinical Nutrition Department, Mahalla Hepatology Teaching Hospital, El-Mahalla El-Kubra 4260010, Egypt
* Correspondence: helrabey@ut.edu.sa

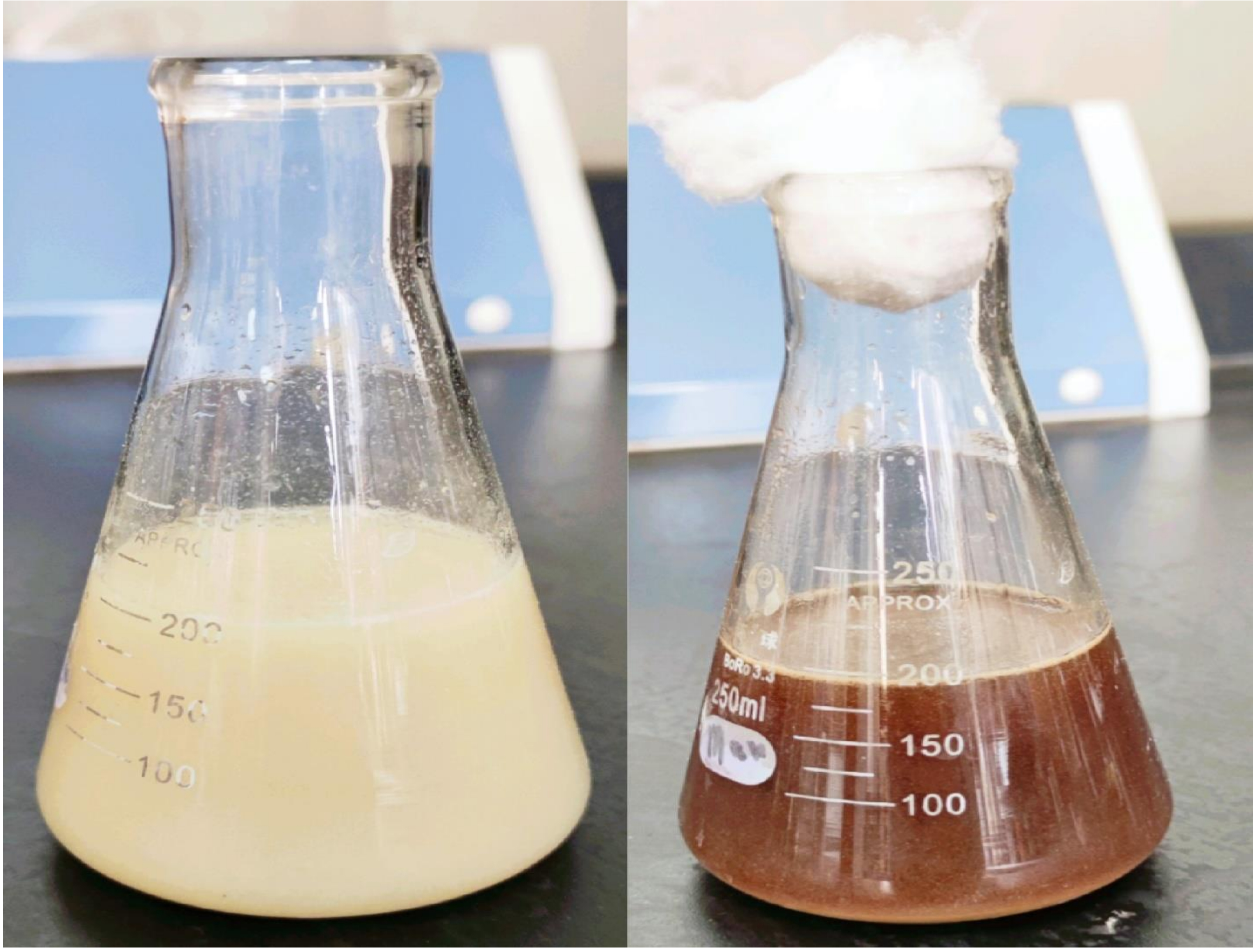

Figure S1. On the left, vanillic acid solution before the addition of silver nitrate. On the right, the color of the vanillic acid solution changed to dark brown after the addition of silver nitrate which indicates the formation of AgNPs.

Table S1. Effect of vanillic acid−loaded silver nanoparticles on serum liver enzymes in CCl_4 liver toxicity−induced rats.

Parameters mg/dl	Statistics	G1 (Negative control)	G2 (Positive control)	G3 (Treated with vanillic)	G4 (Treated with AgNPs vanillic acid)	G5 (Treated with silymarin)
ALT	Mean ± SE LSD0.05 = 12.026	21.33 ± 5.01[a]	77.33 ± 4.06[e]	68.33 ± 2.76[d]	63.00 ± 4.66[c]	53.66 ± 2.13[b]
	T−test	−	11.73***	−11.48***	−21.87***	−9.49***
AST	Mean ± SE LSD0.05 = 10.583	24.33 ± 2.56[a]	90.00 ± 3.84[e]	72.6600 ± 2.55[d]	68.33 ± 2.37[c]	57.33 ± 4.26[b]
	T−test	−	35.43***	−09.85***	−25.25***	−20.72***
ALP	Mean ± SE LSD0.05 = 0.189	133.40 ± 0.10[a]	213.66 ± 0.02[e]	193.00 ± 0.04[d]	182.33 ± 0.04[c]	164.33 ± 0.05[b]
	T−test	−	41.60***	−27.21***	−25.69***	−31.28***
GGT	Mean ± SE LSD0.05 = 5.688	22.33 ± 2.07[a]	67.00 ± 1.52[e]	54.33 ± 2.28[d]	43.00 ± 2.78[c]	33.33 ± 3.31[b]
	T.test	−	11.78***	−06.48***	−07.44***	−09.16***

Data are represented as mean ± SE. T−test values, ***: significant at $p < 0.001$. ANOVA analysis: within each row, means with different superscript (a, b, c, d or e) are significantly different at $p < 0.05$, whereas means superscripts with the same letters mean that there is no significant difference at P<0.05. LSD: least significant difference.

Table S2. Effect of vanillic acid−loaded silver nanoparticles on serum lactate dehydrogenase (LDH), irisin and IL−6 in CCl_4 liver toxicity−induced rats.

Parameters mg/dl	Statistics	G1 (Negative control)	G2 (Positive control)	G3 (Treated with vanillic)	G4 (Treated with AgNPs vanillic acid)	G5 (Treated with silymarin)
LDH (IU/L)	Mean ± SE LSD0.05=11.744	183.83 ± 7.15^{e}	365.33 ± 3.69^{a}	290.00 ± 2.28^{b}	262.10 ± 1.68^{c}	223.00 ± 2.89^{d}
	T−test	−	−7.54***	3.50***	6.94***	6.87***
Irisin (ng/mL)	Mean ± SE LSD0.05=0.305	10.80 ± 0.13^{a}	1.77 ± 0.04^{e}	3.17 ± 0.07^{d}	4.41 ± 0.13^{c}	6.57 ± 0.10^{b}
	T−test	−	00.83***	−1.26***	−1.79***	−3.26***
IL−6 (pg/mL)	Mean ± SE LSD0.05=1.825	40.70 ± 0.45^{e}	90.33 ± 1.13^{a}	78.86 ± 0.49^{b}	64.90 ± 0.60^{c}	56.90 ± 0.70^{d}
	T−test	−	−3.77***	3.58***	1.56***	3.41***

Data are represented as mean ± SE. T−test values, ***: significant at $p < 0.001$. ANOVA analysis: within each row, means with different superscript (a, b, c, d or e) are significantly different at $p < 0.05$, whereas means superscripts with the same letters mean that there is no significant difference at P<0.05. LSD: least significant difference.

Table S3. Effect of vanillic acid−loaded silver nanoparticles on malonaldehyde (MDA) in liver tissues of CCl_4 liver toxicity−induced rats.

Parameters Mg liver tissue/dl	Statistics	G1 (Negative control)	G2 (Positive control)	G3 (Treated with vanillic)	G4 (Treated with AgNPs vanillic acid)	G5 (Treated with silymarin)
MDA nmol/ g. liver tissue	Mean ± SE LSD0.05=1.417	1.78 ± 0.05^{e}	18.23 ± 0.99^{a}	9.63 ± 0.21^{b}	7.90 ± 0.09^{c}	5.60 ± 0.16^{d}
	T−test	T−test	−	−17.01***	11.64***	09.72***

Data are represented as mean ± SE. T−test values, ***: significant at $p < 0.001$. ANOVA analysis: within each row, means with different superscript (a, b, c, d or e) are significantly different at P<0.05, whereas means superscripts with the same letters mean that there is no significant difference at $p < 0.05$. LSD: least significant difference.
